# 

**Published:** 1875-03-15

**Authors:** 


AMERICAN MEDICAL ASSOCIATION.—We hope the profession of the Northwest will remember that the next meeting of the American Medical Association is to be held in Louisville, Ky., commencing on the morning of the first Tuesday in May next. Let every State Medical Society, and every City, County, and District Medical Society entitled to representation in their State Society, see to it that their delegates are appointed, and that they attend the meeting. From information which we have received, we anticipate an unusually interesting and profitable meeting. Assurances of attendance on the part of some of the most eminent men in New York and New England, add to our confidence, that the meeting will be a good one; and also to our desire that the profession in the Northwest should be well represented.

### PAMPHLETS RECEIVED.

MIGRANTS AND SAILORS, Considered in Their Relations to the Public Health, by John M. Woodworth, M.D. and Heber Smith, M.D. Riverside Press, Cambridge. A Reprint from Proceedings of American Public Health Association.

PHYSICIAN'S POCKET CASE-RECORD AND PRESCRIPTION BLANK BOOK, WITH VISITING LIST. Cincinnati: Case Record Company. 35c. each; $3.50 per dozen. A larger office edition similarly arranged, but without the Visiting List, $1.50 each. For sale by Keen, Cooke & Co., Chicago.

This is the most compact and convenient book for the purpose that we have ever seen. It is of convenient size for the pocket, contains about ninety prescription blanks, with stubs for copy of the prescriptions, and record of cases, and a visiting list calculated for thirty patients per week for one month.

---

SCLERITIS SYPHILITICA; Its Pathology, Course and Treatment. By Fred. R. Sturges, M.D. New York: G. P. Putnam's Sons.

---

THE RELATIONS OF THE NERVOUS SYSTEM TO DISEASES OF THE SKIN, by L. Duncan Buckley, M.D. New York: G. P. Putnam's Sons.

---

A RETROSPECT OF THE STRUGGLES AND TRIUMPH OF OVARIOTOMY IN PHILADELPHIA. Annual Address Before the Philadelphia County Medical Society, by Wash. R. Atlee, M.D. Thos. Collins, 705 Jayne Street.

---

PRACTICE FOR SALE.

A Physician's practice, good will, etc., at one of the famous Springs, South. Price, $20,000 cash, or good secured paper. The practice now amounting to upwards of $40,000 actual cash receipts yearly, and increasing. The Physician who wishes to sell, has amassed a competency, and wishes to retire. For full particulars, please address, L. A. GILBERT,

206 La Salle Street,

Chicago, Ill.

*TERMS.— Yearly Subscriptions, Three Dollars, in advance. Single Number Fifteen Cents. All Communications should be addressed to Publishers* MEDICAL EXAMINER, 65 *Randolph Street, corner State, Chicago. Postage, Fifteen Cents per annum.*

Subscribe for your Journals through our office and obtain them at

**CLUB RATES.**

| | Regular Price. | With Examiner. |
|---|---|---|
| The London Lancet (Am. Rep.) | $5 00 | $7 00 |
| Philadelphia Medical and Surgical Reporter, (weekly) | 5 00 | 7 00 |
| " " Times (weekly) | 5 00 | 7 00 |
| New York Medical Record (weekly) | 5 00 | 7 00 |
| " " Journal (monthly) | 4 00 | 6 00 |
| Chicago Medical Journal (monthly) | 4 00 | 6 50 |
| " Journal of Nervous and Mental Disease (quarterly) | 4 00 | 6 00 |

Any other of our home or foreign Journals furnished with THE EXAMINER at ten per cent. discount from the gross subscription price. Address:

**PUBLISHERS MEDICAL EXAMINER,**
65 East Randolph Street, CHICAGO

